# The Dual Functional Reflecting Iris of the Zebrafish

**DOI:** 10.1002/advs.201800338

**Published:** 2018-06-06

**Authors:** Dvir Gur, Jan‐David Nicolas, Vlad Brumfeld, Omri Bar‐Elli, Dan Oron, Gil Levkowitz

**Affiliations:** ^1^ Department of Physics of Complex Systems Weizmann Institute of Science Rehovot 7610001 Israel; ^2^ Department of Molecular Cell Biology Weizmann Institute of Science Rehovot 7610001 Israel; ^3^ Institute for X‐Ray Physics University of Göttingen Göttingen 37077 Germany; ^4^ Department of Chemical Research Support Weizmann Institute of Science Rehovot 7610001 Israel

**Keywords:** biomineralization, guanine crystals, iridophores, photonic crystals, vision, zebrafish development

## Abstract

Many marine organisms have evolved a reflective iris to prevent unfocused light from reaching the retina. The fish iris has a dual function, both to camouflage the eye and serving as a light barrier. Yet, the physical mechanism that enables this dual functionality and the benefits of using a reflective iris have remained unclear. Using synchrotron microfocused diffraction, cryo‐scanning electron microscopy imaging, and optical analyses on zebrafish at different stages of development, it is shown that the complex optical response of the iris is facilitated by the development of high‐order organization of multilayered guanine‐based crystal reflectors and pigments. It is further demonstrated how the efficient light reflector is established during development to allow the optical functionality of the eye, already at early developmental stages.

In the eyes of numerous organisms, including mammals, reptiles, birds, and fish, light is absorbed by photoreceptors in the basally located retina. In order to obtain a high‐contrast image, only light that passes through the pupil and is focused by the lens should reach the retina. Hence, a key function of the iris is to prevent the passage of unfocused light. Irises can be classified either as absorptive, or as reflective or scattering, according to their operating principle with regard to incident light.^1^, ^2^ Many irises of the absorptive type, including in the human eye, contain melanin pigments that efficiently absorb a broad spectrum of visible light.^1^, ^3^ An alternative strategy is employed by numerous marine organisms, in which the outer surface of the iris is highly reflective.^2^, ^4^, ^5^ The reflecting iris of many fish species consists of intracellular crystals that are formed by the nucleobase guanine.^6^, ^7^, ^8^ These guanine crystals are assembled in specialized cells, named iridophores, which are located mainly in the eyes and skin.^2^, ^9^, ^10^ In addition to its function as a light barrier, the iris of fish has a second role of camouflaging the eye by creating a silvery reflection similar to that of fish skin.^2^, ^6^, ^8^ Both these functions require the iris to reflect light efficiently. However, for effective camouflage the iris should reflect light in a highly directional manner,^6^, ^8^ whereas to block light the iris should reflect or scatter light independently of angle or wavelength.^1^, ^3^

Although the dual function of the fish iris has long been known,^2^, ^8^ the underlying mechanism that enables this duality has remained unclear. Here, we unravel the unique ultrastructure of iridophores in the zebrafish iris, which comprises multilayered organization of the guanine‐based crystals. We directly associate the iris crystal organization to both simulated and measured reflectance spectrum. We further show that the structural organization of the developing zebrafish iris is ideally designed to reflect light efficiently at a very early stage of development, which correlates well with the reported high visual acuity of the larva. Mapping of crystal orientations across the entire iris revealed the high‐order crystal organization, which enables the dual function of the iris.

To elucidate iris anatomy and the subcellular organization of the guanine‐based reflectors, we used the zebrafish eye as a model system. In it, the lens is surrounded by a silvery iris, termed *argenta*, which is exposed to direct external light (**Figure** **1**a–c). We first imaged the entire zebrafish eye using micro‐CT (Figure 1d–f), in which the guanine‐based crystal layers of the iris are clearly visible due to their high density. Serial scans showed that the zebrafish iris extends to the lower part of the eyeball, where it forms a reflecting mirror behind the retina, termed the *tapetum lucidum* (Figure 1b–e). The upper, outermost layer of the eye was devoid of the reflecting guanine‐based layer and, instead, was covered by melanin pigments (Figure 1b). In tomographic images of sagittal slices through the dorsal (upper) or ventral (lower) part of the eye, the iris surface appeared as a continuous layer across the entire section (Figure 1e), whereas in a sagittal slice through the center of the eye, the two sides of the iris were separated by the lens (Figure 1f). The use of zebrafish was according to the Weizmann Institute's Institutional Animal Care and Use Committee (#27100516 and #27220516).

**Figure 1:**
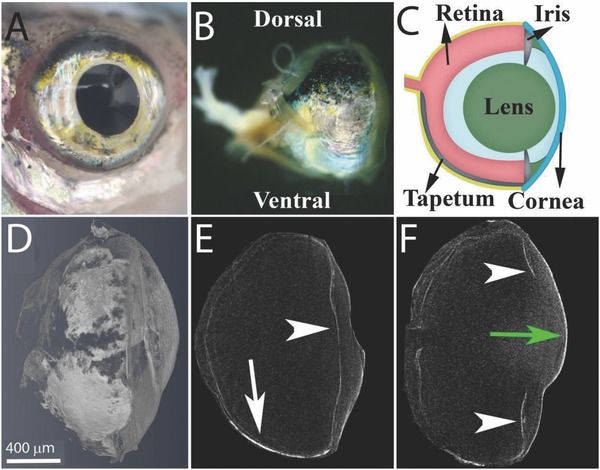
The anatomy of the zebrafish eye. A) A top view of the zebrafish eye showing the silvery iris. B) A side view of the fish eye showing the *tapetum lucidum*, a silvery layer, located at the ventral side of the eyeball and a pigmented layer visible at the dorsal side of the eye. C) A schematic representation of the eye showing the cornea (blue), lens (green), and retina (light red), as well as the guanine‐based reflecting layers, the iris *argenta* and the *tapetum lucidum* (silver). D–F) Micro‐CT images of the zebrafish eye: a volume rendering of the whole eye, a sagittal view of the lower part of the eye, and a sagittal view of the center of the eye. Arrowheads indicate the iris *argentum*, white arrow indicates the *tapetum lucidum*, and green arrow indicates the cornea. Scale bars: 400 µm.

To visualize the organization of the guanine‐based reflectors in the adult zebrafish iris under near‐physiological conditions we used cryo‐scanning electron microscopy (SEM), which allows preservation of intact guanine crystals in their native cellular context (**Figure** **2**a,b). Imaging of coronal sections through the eye revealed that the iris is composed of three different layers: an upper layer of ordered iridophores, a middle layer of disordered iridophores, and a thin layer (≈5 µm) of pigment‐forming melanophores, which lies beneath the two iridophore layers (Figure 2a,e). The cryo‐SEM analysis also showed that the outermost, ordered iridophore layer was composed of monodisperse elongated hexagonal guanine crystals, which were stacked on top of each other (Figure 2a,c and Figure S1, Supporting Information). These crystals displayed an average thickness of 25 ± 7 nm (*n* = 150) and an average cytoplasm spacing of 142 ± 25 nm (*n* = 140). In the adjacent disordered layer, the spacing between crystals was highly variable and their arrangement appeared random, with no preferred orientation (Figure 2a,d). Notably, the disordered iridophore layer was present only in the iris *argenta* and was missing from the *tapetum lucidum (Figure S2, Supporting Information)*.

**Figure 2:**
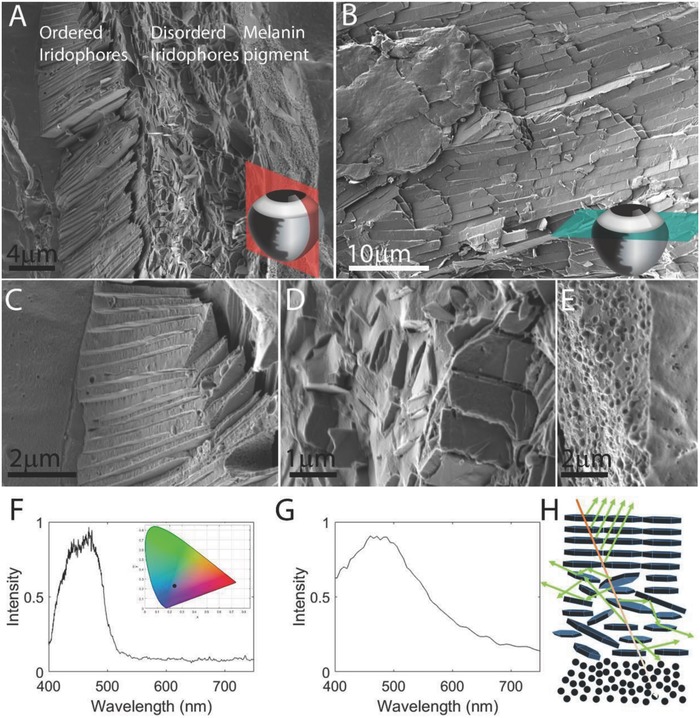
The ultrastructure and optical properties of the iris. A) A coronal section through the iris showing three distinct layers: (i) ordered iridophores, (ii) disordered iridophores, and (iii) a pigmented layer. The section was taken from the periphery of the eye where the iris is curved and, thus, the crystals in the ordered layer are tilted. B) A top view of the iris obtained by a transverse section through the eye shows the elongated guanine crystals packed one next to the other, perfectly tiling the surface of the iris. The anatomical location of sections is illustrated in the bottom‐right corners in (A) and (B). C–E) Higher magnifications of the different iris layers: ordered iridophores, disordered iridophores, and pigmented layer. A–E) Cryo‐SEM images of high‐pressure frozen, freeze‐fractured iris. F) Graph showing measured reflectance from the iris, revealing a broad peak centered around 450 nm. Inset shows the color of reflected light (black dot) plotted on a 1931 CIE chromaticity space diagram. G) Simulated reflectance of the ordered layer calculated based on the data in high‐resolution cryo‐SEM images. H) A sketch illustrating the different paths of light traveling through the multilayered iris. Scale bars: 4 µm (A), 10 µm (B), 2 µm (C,E), 1 µm (D).

We next imaged a horizontal section of the eye (Figure 2b), where the ordered layer was seen as an array of tightly packed crystals perfectly tiling the surface of the iris. The tiling of the crystals was extremely compact, to the extent that it was difficult to separate between iridophores. These results show that in the iris of zebrafish eye, the *argenta* and *tapetum lucidum* form a continuous layer (Figure 1e). Yet, whereas the *tapetum* is composed only from one ordered layer of iridophores (Figure S2, Supporting Information), the *argenta* has a second, disordered guanine‐based layer underlaid by a thin melanosome‐based pigment layer (Figure 2a).

We hypothesized that the three‐layer composition of the iris underlies a complex optical response, which combines wavelength‐dependent light reflection, scattering and absorption. Because impinging light will first hit the reflectors of the outermost layer of the iris, we assumed that the majority of light would be reflected from the ordered layer. To simulate the reflectance of the ordered layer, we analyzed our cryo‐SEM data, namely, the spacing between crystals, using the Monte Carlo transfer matrix method (Supporting Information). The analysis showed that the ordered layer mostly reflects blue‐green light, which peaked at 450 nm (Figure 2g). To correlate our simulated results with actual optical measurements, we used a custom‐built light microscope equipped with a spectrophotometer. Indeed, spectral measurements showed that ≈80–90% of the blue‐green light was reflected from the iris (Figure 2f). The agreement between simulated calculations and optical measurements suggests that the majority of the blue‐green light is indeed reflected from the ordered iridophore layer of the zebrafish iris.

Based on the combined results of ultrastructure imaging, light reflectance simulations and real‐time optical measurements, we propose a model for light traveling through the iris (Figure 2h). The ordered iridescent layer reflects ≈80–90% of the impinging blue‐green light. The residual light that passes through the first layer is scattered by the disordered layer. Finally, the thin pigmented layer likely absorbs any light passing the two guanine‐based layers, including light that is forward‐scattered from the disordered layer. In addition, the scattering of light by the disordered layer, as it likely introduce an angular spread to the incoming light, increases the probability for light to be absorbed by the melanin pigment.

Zebrafish reproduce by external fertilization, which means that the offspring must be self‐sufficient very early in life. High visual acuity is pertinent to the survival of free‐swimming zebrafish, which rely on visual stimuli for prey capture.^11^, ^12^ Previous studies showed that iridophores in zebrafish iris could be detected as soon as 3 dpf.^5^ We therefore predicted that the zebrafish iris would be highly functional at early developmental stages. To test this hypothesis, we analyzed the optical properties, iridophore ultrastructure, and crystal organization of the zebrafish iris at different stages of development. Light microscopy revealed that iridophores were indeed clearly visible in the iris already at 3 d postfertilization (dpf), where they intially occupy the inner circumferential layer, lining the inner radius of the iris around the lens (Figure S3, Supporting Information). At 7 dpf, iridophores covered only about half of the iris surface (**Figure** **3**a). At 14 dpf, the iridophores occupied a larger area of the iris; however, large dark patches showing the underling pigmented layer, were still visible. By 21 and 28 dpf, the iridophores covered nearly the entire surface of the iris (Figure 3c,d).

**Figure 3:**
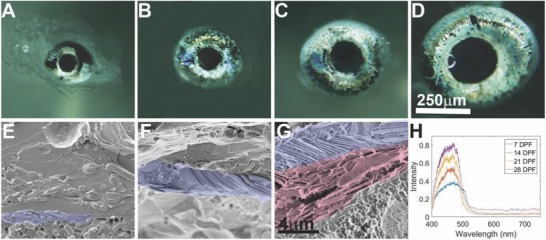
Iris development. Iris morphology, ultrastructural and optical properties were examined during development at 7 (A,E), 14 (B,F), 21 (C,G), and 28 (D) dpf. A–D) Light microscope images of the larva iris at different stages. E–G) Cryo‐SEM images of high‐pressure frozen, freeze‐fractured larva iris show the ultrastructure of the developing iris. Whereas at 7 and 14 dpf only the ordered layer is seen, by 21 dpf a second, disordered layer starts to appear. Ordered iridophores (purple) and disordered iridophores (light red) are shown in pseudo‐colors. H) Graph showing measured reflectance of irises from 7, 14, 21, and 28 dpf larvae, revealing a peak reflectance centered around 450 nm. Scale bars: 250 µm (A–D), 4 µm (E–G).

To elucidate the development of the iris ultrastructure, we performed cryo‐SEM imaging at corresponding development stages. We found that at 7 dpf, the guanine crystals in the clearly visible iridophores were arranged in relatively ordered parallel stacks, similar to the adult iris (Figure 3e). However, at this stage, only one layer of iridophores was visible. Moreover, individual crystals were smaller than those observed in adults and, in certain cases, the membranes engulfing the crystal seemed less tight than in adults (Figure 3e). At 14 dpf, still only one layer of iridophores was present, but the crystal stacks were more ordered compared to 7 dpf (Figure 3f). By 21 dpf, iris thickness increased considerably and the second layer of iridophores was sometimes visible (Figure 3g), usually in the areas closest to the lens (Figure S4, Supporting Information). At this age, guanine crystals and iridophore ultrastructure resembled those of adult fish, suggesting that the iris is already fully functional as a reflecting light barrier.

To determine the functionality of the iris during development, we measured the reflectance spectrum at corresponding stages (Figure 3h). Despite the incomplete crystal layers and iridophore coverage at larval stages, a wide reflectance peak centered on 450 nm wavelength was measured from a very early stage (7 dpf) until adulthood (Figure 3h). The reflectance intensity increased with age, averaging at ≈40% for 7 dpf larva and reaching ≈80% by 28 dpf, very close to the levels observed in adults. Taken together, these results indicate that the larval iris functions as an efficient light‐barrier already at 7 dpf, and that this initial functionality is preserved and optimized throughout eye development. These findings are consistent with the reported high visual acuity of very early stage zebrafish larvae.^11^, ^12^

Having found that light is reflected mostly from the outermost ordered iridophore layer, we hypothesized the existence of an intrinsic, high‐order organization of crystal stacks within this layer. To examine this, we sought to map the orientation of the guanine crystals stacks across the entire iris. Because the adult zebrafish iris is composed of millions of crystals extending across several square millimeters, mapping crystal orientations accurately using high‐resolution imaging techniques such as cryo‐SEM was not feasible, whereas modalities for imaging relatively large areas, such as micro‐CT, would not provide sufficient resolution to determine the orientation of individual stacks. Therefore, we opted to use scanning X‐ray diffraction combined with a microfocused synchrotron beam. Thus, the X‐ray beam continuously scanned across the sample and the diffraction patterns were collected with a single‐photon counting flat panel detector at regular time intervals. The scanning speed was set such that a diffraction pattern was collected every 4 µm. Thus, a single scan could contain over 400 000 diffraction patterns, covering the entire surface area of the iris at an overall scan time of about 1 h per sample. To analyze the data in a standardized, robust, and model‐independent fashion, we used a custom Matlab‐based principal component analysis (PCA) based on Bernhardt et al.^13^ as part of a Nano‐diffraction toolbox described by Nicolas et al.^14^ (Supporting Information). Using this PCA algorithm, we extracted the anisotropy and orientation of light scattering and, subsequently, inferred the orientation of the crystals.

Based on our transmission electron microscopy (TEM) studies (Figure S5, Supporting Information), the crystals in the fish iris are anhydrous β‐guanine (100) crystal plates, (**Figure** **4**c). Thus, the (100) plane is in diffraction for crystals that are oriented almost edge‐on to the beam (see Figure 4a color bar for crystal orientation). Mapping the anisotropy and orientation of the (100) diffraction plane revealed two regions of crystal stacks, an inner layer that surrounded the lens and an outer layer surrounding the outer radius of the eye (Figure 4a). The crystals in the two layers were positioned tangent to the iris circumference, lining the top section of the eye. Remarkably, the orientation of the crystal stacks at the inner and outer radii followed the curvature of the iris (Figure 4a). In the outer layer, the crystals extended up to the point where the eye is protruding out from the fish head, suggesting that they prevent light that impinges upon the eye from the side from distorting the fish vision (Figure 4f and Figure S6, Supporting Information). Accordingly, the crystals at the inner layer, which surrounds the lens, would reflect light that is impinged upon the lens sideways, and thus limit the angular acceptance of the eye, leading to a reduction in spherical aberrations. This inner circumference layer may also provide a tighter seal for light at the interface between the lens and the iris (Figure 4a).

**Figure 4:**
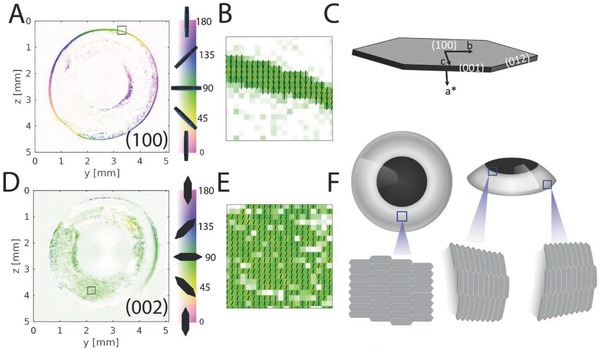
Synchrotron‐based X‐ray diffraction imaging. The orientation of the different crystal planes was mapped across the entire iris using scanning X‐ray diffraction combined with a microfocused synchrotron beam. A,D) Maps showing the orientations (degrees) of the (100) and (002) guanine crystal diffraction planes, respectively. The color gradient (right bar) from white to saturated color corresponds to the respective low and high anisotropy levels. B,E) Magnifications of the squared regions marked in (A) and (D), respectively. C) Schematic illustration of fish guanine crystal, showing the relations between crystal morphology and the crystallographic axes and faces. F) Schematic illustration showing the orientations of the guanine plate‐like crystals at different regions of the eye.

The perpendicular (002) plane should only be in diffraction for crystals that are positioned almost perpendicular to the beam (Figure 4c). Mapping the (002) diffraction plane across the iris showed that the orientation of most of the crystals was parallel to the iris surface (Figure 4d), suggesting that they would efficiently reflect light impinging on the surface (Figure 4f). Due to the curvature of the iris close to its periphery, the crystal were tilted and were thus out of diffraction. Nonetheless, these regions are still tiled by crystals, as observed by mapping the diffraction of all the different crystal planes, regardless of their orientation (Figure S7b, Supporting Information).

Remarkably, the crystals that tile the surface of the iris were aligned throughout the entire iris, covering several square millimeters in adult fish. This co‐orientation of crystals across tens of thousands of cells suggests that the concerted organization of crystals is highly regulated. Presumably, cell communication between iridophores facilitates orienting the crystals across the iris in a synchronized manner. Such coordinated cell communication mechanism was previously reported for other types of pigment cells during zebrafish color pattern formation.^15^ Light reflection from a flat layer of co‐orientated crystals could be analogous to the specular reflection from a flat mirror, which is characterized by a high degree of directionality. Indeed, Fourier transform reflection analysis indicated that reflection from the iris is highly directional (Figure S8, Supporting Information).

In zebrafish, the eyes are located on both sides of the head such that the iris surfaces are at an oblique angle to impinging sunlight. Thus, sunlight should be reflected from the iris at an obtuse angle, preserving the downwelling direction of the light. This may be beneficial for camouflage in shallow waters, where upwelling iridescence is relatively minute.^16^ Thus, light reflected or scattered in any direction other than downwelling would be highly conspicuous, revealing the location of the fish to potential predators. Furthermore, the co‐orientation of the crystals may allow perfect surface tiling and, thus, minimize surface defects. Such defects may lead to transmission of light through the iris and onto the retina, reducing the contrast of the obtained image.

Having both an ordered and a disordered layer enables the dual functionality of the iris, an efficient light barrier that also provides camouflage to the normally black uveal tract. The first ordered layer is an efficient directional light reflector and, at most angles, incident light will be reflected efficiently. However, at very obtuse angles, at which light is almost parallel to the reflectors (e.g., sunlight scattered by an object in the water), or when light is at the red edge of the visible spectrum, the efficiency of the reflectors is reduced dramatically. Having a second layer of varying crystal orientations could provide a solution to this problem. A second functional benefit for having two iris layers relates to the spectral environment of the eye. In the marine environment, the longer wavelengths are rapidly absorbed in the water, leaving mostly blue‐green light.^16^, ^17^ Furthermore, the opsins in the zebrafish retina are much more sensitive to shorter wavelengths than to longer ones.^12^, ^18^ Thus, the iris should be very efficient in reflecting shorter wavelengths, while still being able to reflect lower levels of longer wavelengths. The two superimposed layers in the iris provide an elegant solution to these requirements. The ordered layer has a peak reflectance for shorter wavelengths and the disordered layer scatters most wavelengths, but to a lesser extent. The organization of the plate‐like crystals in the ordered layer changes considerably in different regions of the iris. The crystals in the center of the iris are parallel to the iris surface, whereas at the edges they are perpendicular to the surface. This high‐order organization allows the iris to efficiently block laterally impinging light and reduces spherical aberration. As mentioned, the co‐orientation of the crystals allows for flawless tiling across the iris surface, thereby providing a tight seal for light. Interestingly, in certain albino fish the peripheral rods evade light damage although the inner iris epithelium lacks melanin, suggesting that the iris *argenta* also provides sufficient light screening to protect these photoreceptors.^19^

Our analyses of the developing iris may provide a functional explanation for the evolutionary choice of a reflective rather than absorptive iris in fish. The diameter of the 5 dpf larval eye is ≈150 µm, increasing up to ≈350 µm at 21 dpf. Taking into account the volume of the other eye parts, the space that the iris may occupy is very limited. An ordered reflecting layer is compact and, thus, advantageous under these conditions. Indeed, we found that the ordered reflecting layer, which is only ≈3 µm thick, provides about 60% reflection of the blue‐green light. In comparison, a melanin‐based iris would require a layer of about 15 µm in thickness to absorb a similar amount of light.^20^

Finally, the ordered crystal layer, which mostly reflects blue‐green light, is advantageous because these wavelengths are the most abundant in the marine environment. The increase in iris reflectivity with age is mostly due to increase in the coverage area by iridophores, but probably also due to the thickening of the ordered reflecting layer. The thin layer of ordered reflectors blocks a sufficiently broad spectrum of light and should allow the developing larvae to form an image, albeit of low contrast, at a very early age. The second disordered layer, which develops later, scatters a broader spectrum of light and, thereby, enables the formation of clearer images of higher contrast. This developmental strategy enables the eye to become functional at a very early stage and remain functional throughout development, as iris efficiency increases.

The fish iris is an exquisite example of nature's remarkable engineering, where the specialized iridophores produce an efficient light reflector made of guanine‐based crystals. We provide a physical mechanism for the dual functionality of the iris, an efficient light barrier and a camouflage for the fish eye. The complex optical response of the iris is achieved by a combination of wavelength‐dependent light reflection from the ordered layer, scattering by the disordered layer, and absorption by the pigmented layer. The high‐order organization of the reflecting layer both optimize the light reflection properties of the iris, stops laterally impinging light, and provides the eye with camouflage. The sophisticated strategy evolved by fish to produce the dual functionality of the iris may serve as an inspiration for the construction of artificial photonic structures with similar complex optical properties.

## Conflict of Interest

The authors declare no conflict of interest.

## Supporting information

Supplementary
