# Supplementary material for: The Dual Functional Reflecting Iris of the Zebrafish
Source: Adv Sci (Weinh). 2018 Jun 6;5(8):1800338. doi: 10.1002/advs.201800338 (PMC6097150; doi:10.1002/advs.201800338)
Supplement: Supplementary file 1 — Supplementary [file ADVS-5-1800338-s001.pdf]

Copyright WILEY-VCH Verlag GmbH & Co. KGaA, 69469 Weinheim, Germany, 2018.

ADVANCED SCIENCE
Open Access

Supporting Information

for *Adv. Sci.,* DOI: 10.1002/advs.201800338

The Dual Functional Reflecting Iris of the Zebrafish

Dvir Gur, Jan-David Nicolas, Vlad Brumfeld, Omri Bar-Elli, Dan Oron, and Gil Levkowitz**

Supporting Information

The Dual Functional Reflecting Iris of the Zebrafish

Dvir Gur, Jan-David Nicolas, Vlad Brumfeld, Omri Bar-Elli, Dan Oron*, Gil Levkowitz*

Experimental section

Zebrafish lines and maintenance: Zebrafish were raised and bred according to the Weizmann Institute Animal Care and Use Committee (IACUC). The zebrafish lines used were either AB or TL wild types.

Chromatic imaging: High-quality color images were collected using an inverted microscope (Eclipse Ti-U, Nikon) equipped with a Nikon high-definition color camera head (DS-Fi2, Nikon) or using a Zeiss Axioplan microscope (Zeiss, Jena, Germany).

Cryo-scanning electron microscopy (Cryo-SEM): For adult zebrafish, fixed eyes were embedded in 3% agar using PBS as the medium. Coronal or transverse sections were cut at a thickness of 200 µm using a vibratome (VT1000-S, Leica). For zebrafish larvae, either fixed or fresh sacrificed larvae (5-28 dpf) were used. The sections or larvae were then sandwiched between two metal discs (3 mm diameter, 0.1 mm cavities) and cryo-immobilized in a high-pressure freezing device (HPM10; Bal-Tec). The frozen samples were mounted on a holder under liquid nitrogen and transferred to a freeze-fracture device (BAF60; Bal-Tec) using a vacuum cryo-transfer device (VCT 100; Bal-Tec), where they were coated with a 4-nm-thick layer of Pt/C. Samples were then observed by high-resolution SEM (Ultra 55, Zeiss) using secondary electron/backscattered electron and an in-lens detector, maintaining the frozen-hydrated state by using a cryo-stage operating at a working temperature of −120°C. Measurements of crystal thickness and cytoplasm spacing were taken from the cryo-SEM micrographs.

X-ray micro-CT: Micro-CT scans were performed using a Micro XCT-400 (Zeiss X-ray Microscopy, California, USA). Whole zebrafish eyes, including the optic nerve, were placed in a plastic pipette tip, which had been sealed by melting using a flame. To prevent dehydration of the sample, the tips were partially filled with water and the eye was held above the water in a saturated water vapor atmosphere. X-ray micro-CT measurements were

performed on a total of 5 whole, hydrated zebrafish eyes. The tomographic volumes were obtained by taking 1100 projections over 180° at 30 KV and 150 µA, and the isotropic voxel size in the reconstructed volume was 2.2 µm.

Reflectivity measurements: The reflectivity of the zebrafish iris was measured on either fixed or recently removed fresh eyes, which were immersed in PBS and placed underneath a cover slip held in place with silicon grease. The eyes were positioned such that the iris was facing directly upwards. Impinging light was approximately normal to the objective lens. Reflectivity was measured as described in detail in [10] with the exception of using a Shamrock 303i spectrometer (Andor) equipped with an iDus 420 CCD camera and cooled to -70°C . Briefly, we used a custom-built microscope consisting of a microspectrophotometer, two CCD cameras, and a high numerical aperture objective, which enabled imaging the iris while obtaining both the reflectance spectrum and the Fourier transform of the reflectance for the same location in the sample. The light source was a halogen lamp coupled to an optical fiber, which guided the light into the microscope.

Imaging was first used to determine the correct focal point for the light source. Then the reflectance spectrum was used to determine the reflectance intensity, which was normalized to the reflectance of a silver mirror. Light was then imaged through a beam splitter onto the back aperture of an objective (Olympus, UPLSAPO 60XW, NA 1.2). The objective was used both to illuminate a wide area (~250 µm in width) and to collect the scattered light. The collected light was directed to one of three different paths by a set of folding mirrors. In the first path, the sample was imaged onto a CCD camera (Mintron, MTV 13 V5Hc). In the second path, the Fourier transform of the scattered light was captured by imaging the back aperture of the objective onto a similar CCD camera. In the third path, the light was collected and coupled into a fiber, which guided the light into a spectrometer. The sample was placed on top of translational stage and goniometer, such that both its position and orientation could be controlled.

Reflectivity simulations: The reflectivity spectrum was simulated based on crystal thicknesses and spacing obtained from cryo-SEM images using a Monte Carlo transfer matrix calculation, as described in detail in the supporting information of [S1]. In brief, the percentage of reflectivity was calculated by averaging 500 runs, assuming normal incident light. Each layer

was characterized by two variables: n_j, a refractive index, and d_j, which is the layer thickness randomly picked from the experimental distribution. Thus, for each layer we defined the following 2 × 2 matrix:

$$m_j = \begin{pmatrix} \cos\beta_j & -\frac{i}{n_j}\sin\beta_j \\ -in_j\sin\beta_j & \cos\beta_j \end{pmatrix} \quad \text{where} \quad \beta_j = \frac{2\pi}{\lambda} n_j d_j$$

The set of k double layers was characterized by an overall reflectivity 2 × 2 matrix:

$$M_j = \prod_{j=1}^{j=2k} m_j$$

The reflectivity was extracted from the following equation:

$$R = \left| \frac{(m_{11}+m_{12})-(m_{21}+m_{22})}{(m_{11}+m_{12})+(m_{21}+m_{22})} \right|^2$$

The refractive index of the guanine crystal plates was taken as 1.83, which was the refractive index in the direction of the impinging light. The weak dependence of the refractive index on wavelength was neglected, assuming that all the interfaces, i.e., inside a crystal stack and between stacks, were parallel. We also assumed no correlation between the crystal spacings within a single crystal stack.

Transmission electron microscopy (TEM) imaging: To extract the guanine crystals, eyes were embedded in 7% agar and cut into 100 µm horizontal sections using a vibratome. The sections were then homogenized and the crystals were concentrated using centrifugeation. A suspension of the crystals in DDW was then removed and a drop was applied to a glow-discharged carbon-coated, copper TEM grid. The suspension was allowed to settle for 30 seconds and were then blotted. The TEM grids were observed using an FEI Tecnai T12 TEM operated at 120 kV. Images and diffraction patterns were recorded on a Gatan OneView camera using imaging and diffraction modes respectively. The observed electron diffraction patterns of the crystals (in set in Fig. S5) correspond to anhydrous β-guanine[S2].

Synchrotron XRD measurements: The scanning XRD measurements were carried out at beamline ID13 of the European Synchrotron Radiation Facility (ESRF) in Grenoble, France. The beam was monochromatized using a Si(111) channel-cut monochromator to an energy of 13.9 keV. Compound refractive lenses were used as a focusing system that yielded a focal spot size of 2 × 2 µm^2. The sample was placed in the focus of the beam and the detector was

positioned 149.25 mm behind the focal plane. The beam center, detector tilt angles and the sample-to-detector distance were determined using Al2O3 as calibrant. Diffraction patterns were collected at high frame rates using the single-photon counting Eiger 4 M detector (Dectris, Switzerland). We were able to acquire diffraction images with an exposure time of 50 ms. The samples were scanned in a continuous movement through the x-ray beam. The 'step size' corresponding to the interval traveled during a single exposure was 2 µm along the horizontal (y) and vertical (z) direction.

Analysis of XRD data: Data correction steps were necessary, before scattering data could be analyzed. First, invalid detector pixels were masked such that their respective value were not taken into account in the analysis. Secondly, the absorption of a semi-transparent capillary used as a beamstop holder was corrected for by pixel wise multiplication with a correction matrix. Thirdly, background was subtracted from each scattering pattern to base the PCA analysis solely on the scattered intensity due to crystalline reflections. Lastly, to reduce data load and to improve the speed of calculation, the PCA analysis was performed only on a range of q-values, centered on the required respective (100), (012) and (002) reflection with a width of $\Delta q = 0.2$ nm^{-1}.

Following the approach described by Bernhardt et al. [13] the scattering distribution is treated as a probability density function for the distribution of photons. To retrieve the eigenvectors of the scattering distribution, the covariance matrix C of the distribution of the wave vector components q_y and q_z in the detection plane is diagonalized. C is defined as

$$C = \begin{pmatrix} Var(q_y) & Cov(q_y, q_z) \\ Cov(q_z, q_y) & Var(q_z) \end{pmatrix}$$

The principal direction of scattering is thereby given by the largest of the two corresponding eigenvectors v_1 and v_2. The length of the eigenvectors (the variance) is given by the eigenvalues λ_1 and λ_2. One can thereby define a dimensionless parameter, the anisotropy of the scattering:

$$\omega = \frac{\lambda_1 - \lambda_2}{|\lambda_1 + \lambda_2|}\,.$$

A value of 1 would thereby correspond to a single scattering direction, while a value of 0 would correspond to a perfectly isotropic scattering.

Based on our analysis, not all of crystals are aligned co-axially with respect to each other as suggested by the anisotropy of the scattering (< 0.2 for the (012) and (002) crystal planes; < 0.5 for the (100) crystal plane, see Fig. S5). The relativley low anisotropy of the

scattering is probably due to the underlying disordered iridiphore layers, which reduces the levels of anisotropy . The anisotropy maps corresponding to Figs. 4A and 4D are shown in Figs. S5 C and D, respectively. Disregarding a particular reflection, one should keep in mind, that at any scan point from within the iris, at least one of the crystal planes meet the diffraction condition. This is shown in the map shown in Fig. S5 B which integrates the scattered intensity that is above a manually chosen threshold.

Several structural parameters can be extracted from a single scattering pattern. It is therefore informative to use multiple representations of the scattering data. First, in Fig. S5 A we show the scattered intensity integrated within the 10.5 nm^{-1} and 11.5 nm^{-1}. The q-range was chosen based on the fact that within this range, no reflections occur. We hereby obtain a dark field contrast that is solely based on the scattering of the isotropic sample matrix and solution. In this contrast, one can for example observe trapped air bubbles in the sample preparation. The overall distribution of crystals in the sample can be obtained by integrating the scattered intensity above a manually chosen threshold that discriminates between Bragg reflections and background scattering. An example is shown in Fig. S5B. Furthermore, the three Bragg reflections can be clearly seen in maximum intensity projections of the entire dataset, see Fig. S5E. To generate a maximum intensity projection, a given pixel with index (i,j) is assigned the maximum value of all pixels with index (i,j) in the entire data set. Clearly, all reflections can hereby be visualized while in an average scattering pattern, a single reflection would be averaged out and barely visible. For comparison, a single diffraction pattern is shown in Fig. S5F.

In addition to the contrasts presented so far, one can furthermore radially integrate a single scattering pattern within a given q-range to yield a one-dimensional representation of the intensity as a function of azimuthal angle I(phi). We have performed a radial integration on the (100) reflection for each scan point. We then calculated the normalized cross-correlation of the radial intensity of each scan point with its next neighbors. We found that adjacent scan points are well correlated over a distance of 4 µm (distance between two scan points), especially, where the anisotropy was high. An overview over the entire sample as well as a zoom region is shown in Figs. S5 G and H, respectively.

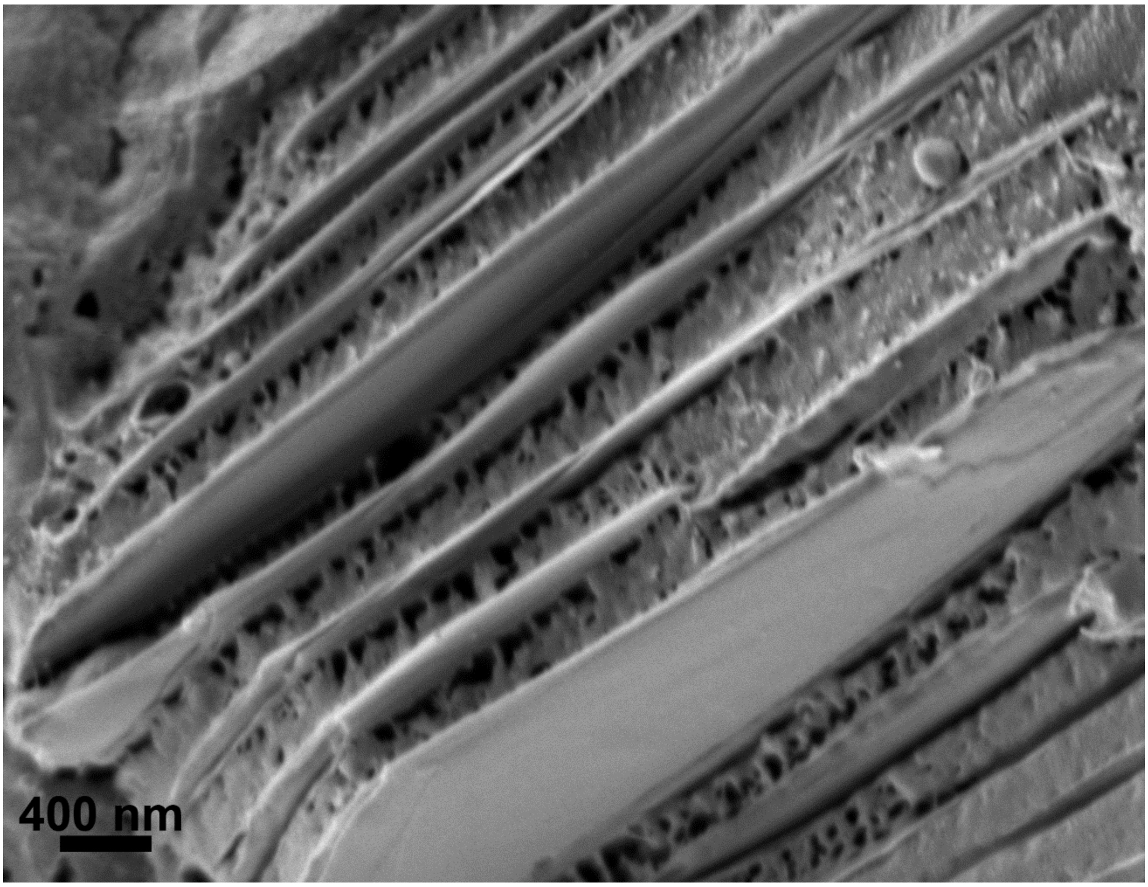

Figure S1. Cryo-SEM image of the ordered iridophore layer in an adult zebrafish iris. Image of a freeze-fracture adult iris shows the parallel stack of thin guanine crystals separated by thicker cytoplasm spacings.

(A), 200 µm (B).

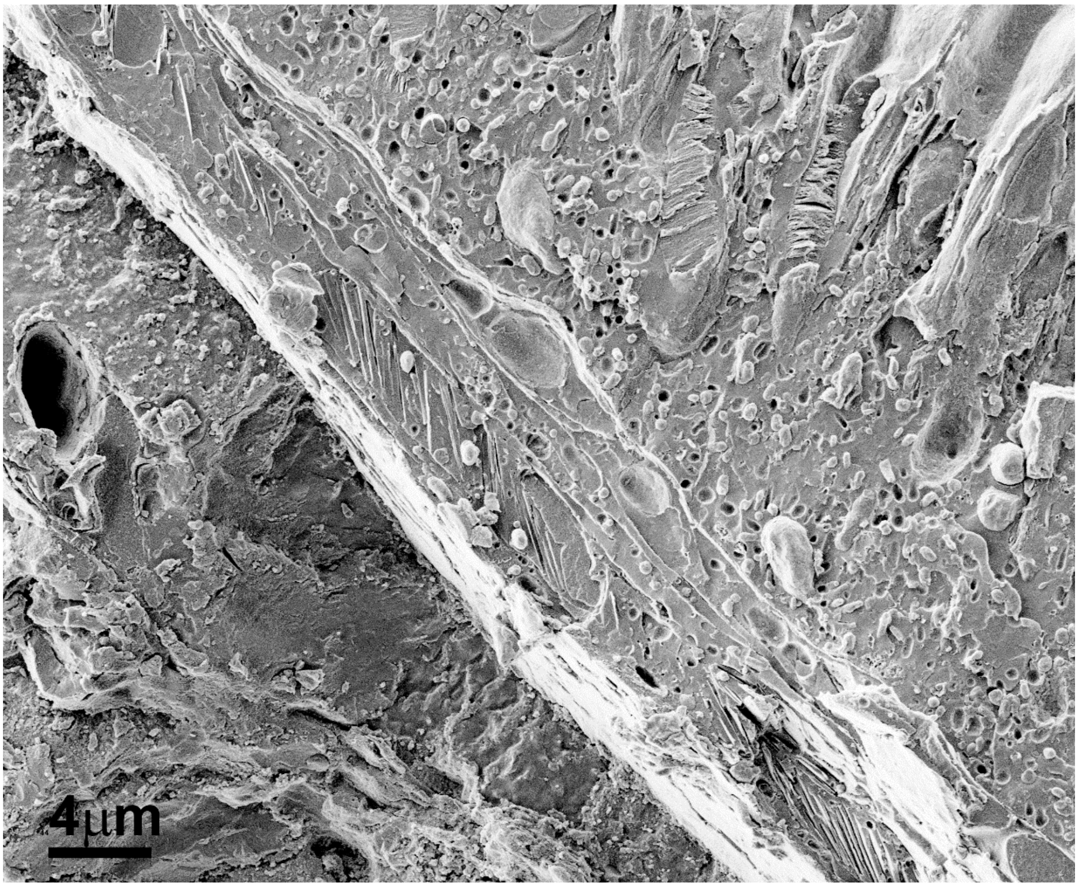

Figure S2. Cryo-SEM image of the tapetum layer in a 21dpf zebrafish eye. Image of a freeze-fracture adult eye shows that the tapetum layer is composed only from one ordered layer of iridophores.

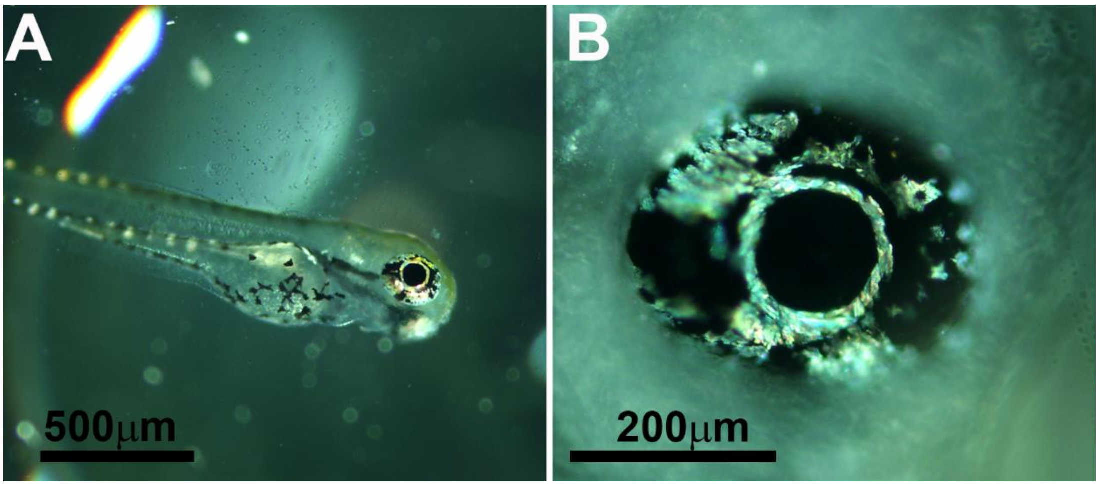

Figure S3. Light microscopy images of 3 dpf zebrafish larvae. (A) A complete larva. (B) Magnification of the eye. Already at this stage, the iridophores in the larval iris are clearly visible. The iridophores surrounding the lens seem to be the first to form. Scale bars: 500 µm (A), 200 µm (B).

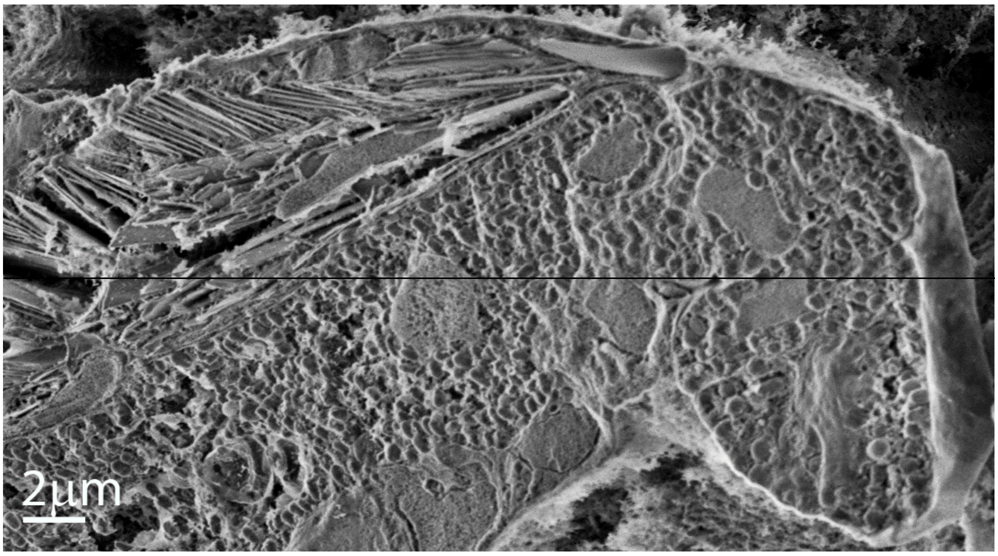

Figure S4. Cryo-SEM image of the zebrafish iris at 21 dpf. Image of a freeze-fractured iris shows the area adjacent to the lens, where two layers of iridophores are visible. Scale bar: 2 µm.

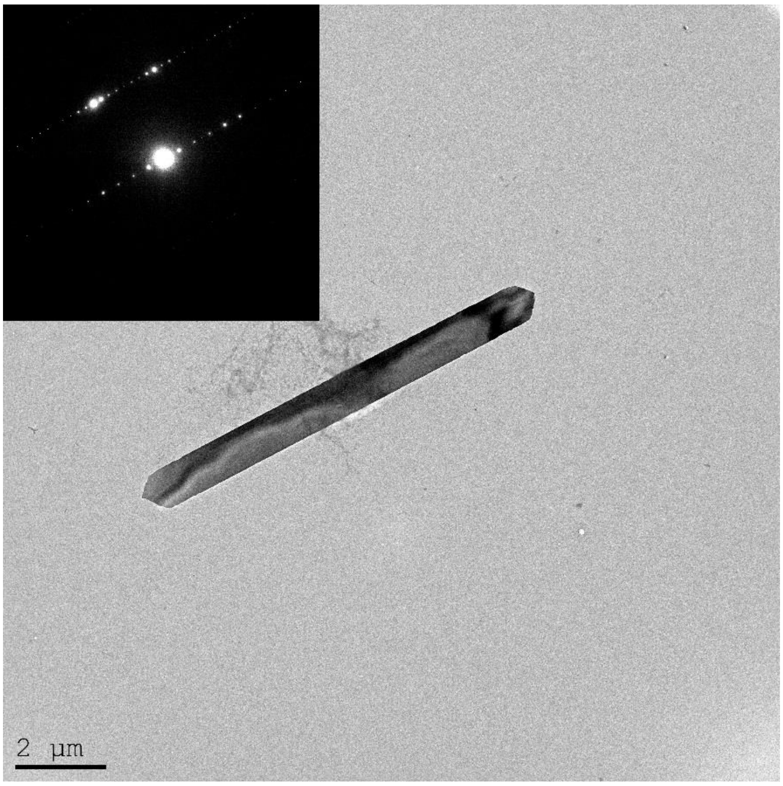

Figure S5. TEM images of a single guanine crystal. A TEM image showing a single crystal extracted from the zebrafish iris and its electron diffraction (inset). The electron diffraction correspond to anhydrous β-guanine.

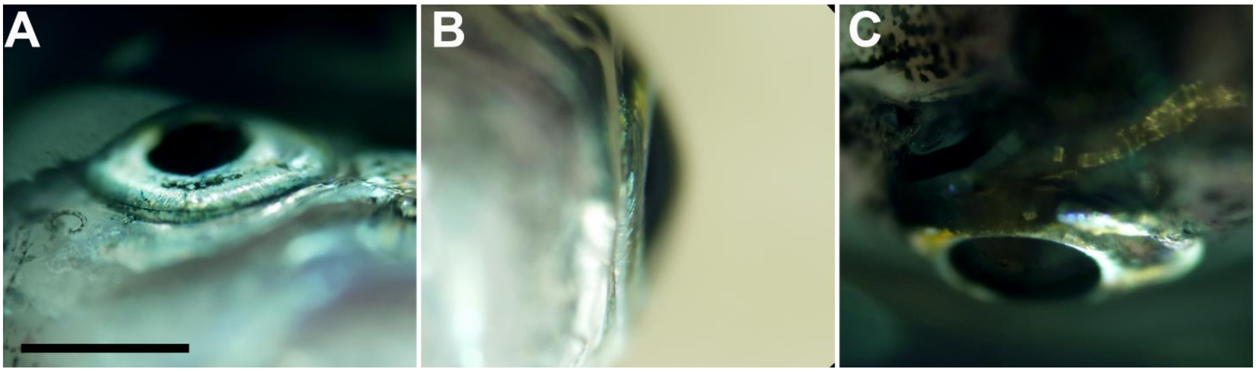

Figure S6. Light microscope images of the outer iridophores surrounding the eye. This layer extends to the point where the eye is protruding out from the fish head. A) Ventral view of the eye. B) A side view from posterior to anterior. C) A dorsal view of the eye.

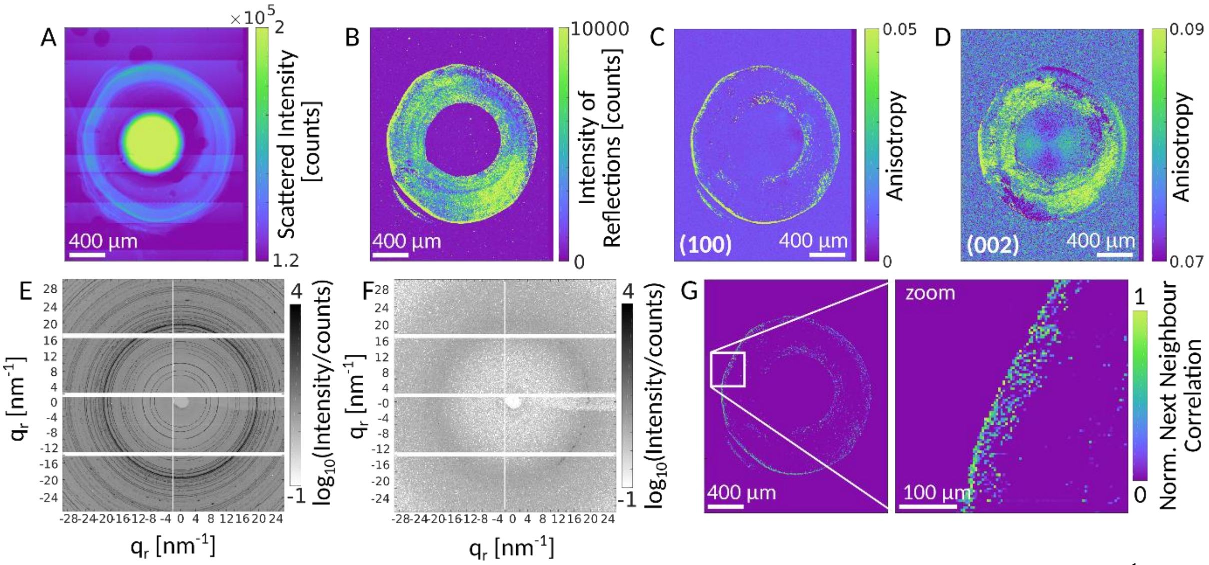

Figure S7. Analysis of diffraction patterns. (A) Integrated intensity between 10.5 nm^{-1} and 11.5 nm^{-1}. (B) Sum over all pixels with an intensity greater than 50 counts. (C,D) Anisotropy of the (100) and (002) reflections. (E) Maximum intensity projection and (F) Isolated diffraction pattern. (G) Correlation of the radial intensity of the (100) reflection with data from its next neighbors. A zoom is shown on the right.

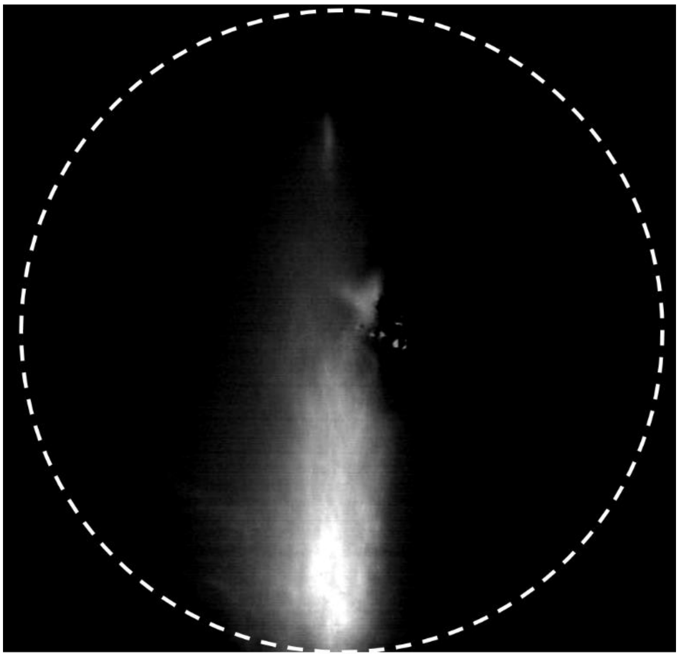

Figure S8. Fourier transform image of an adult zebrafish iris reflectance. A microscopic image showing the highly directional reflection of an adult zebrafish iris.

Supporting information references:

[S1] D. Gur, B. Leshem, M. Pierantoni, V. Farstey, D. Oron, S. Weiner, L. Addadi, J. *Amer. Chem. Soc.* **2015**, *137*, 8408.

[S2] a) A. Hirsch, D. Gur, I. Polishchuk, D. Levy, B. Pokroy, A. J. Cruz-Cabeza, L. Addadi, L. Kronik, L. Leiserowitz, *Chem. Mat.* **2015**, *27*, 8289. b) D. Gur, M. Pierantoni, N. Elool Dov. A. Hirsch, Y. Feldman, S. Weiner, L. Addadi, *Cryst. Growth Des.* **2016**, *16*, 4975.
